# Magnetic field interactions of smartwatches and portable electronic devices with CIEDs – Did we open a Pandora’s box?

**DOI:** 10.1016/j.ijcha.2022.101122

**Published:** 2022-09-08

**Authors:** Patrick Badertscher, Céline Vergne, Corentin Féry, Diego Mannhart, Thomas Quirin, Stefan Osswald, Michael Kühne, Christian Sticherling, Sven Knecht, Joris Pascal

**Affiliations:** a Cardiovascular Research Institute Basel, University Hospital Basel, University of Basel, Basel, Switzerland; b Institute for Medical Engineering and Medical Informatics, School of Life Sciences, University of Applied Sciences and Arts Northwestern Switzerland, Muttenz, Switzerland; c Icube Laboratory, University of Strasbourg - CNRS, Strasbourg, France

**Keywords:** CIED, Magnetic field, Interaction, Portable electronic devices, Smartwatch, Apple Watch

## Abstract

**Introduction:**

Magnetic interaction of portable electronic devices (PEDs), such as state-of-the art mobile phones, with cardiovascular implantable electronic devices (CIEDs) has been reported. The aim of the study was to quantify the magnetic fields of latest generation smartwatches and other PEDs and to evaluate and predict their risk of CIED interactions.

**Methods:**

High resolution magnetic field characterization of five smartwatches (Apple Watch 6/7, Fitbit Sense, Samsung Galaxy 3, Withings Scanwatch) was performed using a novel magnetic field camera. *Ex vivo* measurements of the minimal safety distance (MSD) at which no mode switch can be observed were performed between 11 PEDs and six representative CIEDs.

**Results:**

Maximal 1 mT distances ranged between 10 mm (Withings) and 19 mm (Fitbit and AppleWatch), and 1 mT volumes between 6 cm^3^ (Withings) and 19 cm^3^ (Fitbit). All these measures were observed only for the back side of the smartwatches. While most smartwatches with measured 1 mT distance < 15 mm posed low *ex vivo* interaction within a distance of < 10 mm, PEDs such as electronic pens and in-ear-headphones with measured 1 mT distance > 15 mm showed device interaction up to > 15 mm. Linear regression analysis showed a linear relationship of the MSD with 1 mT distance (B coefficient: 0.46; 95 %-CI: 0.25–0.67, p < 0.001).

**Conclusion:**

Smartwatches are safer compared to other PEDs such as electronic pens or in-ear headphones with regards to CIED interaction. With a standardized magnetic field camera, the risk assessment of CIED interaction of novel PEDs is feasible.

## 1 Introduction

With their rising availability, smartwatches as one group of the growing field of portable electronic devices (PEDs) play an increasing role in cardiology due to its capability to acquire single-lead electrocardiograms (ECGs) and to detect atrial fibrillation (AF). With the admission of these single-lead ECGs for the documentation and screening of AF in the latest ESC guidelines [1], their use will most likely increase in future not only in younger individuals. Cardiac patients may by carriers of cardiovascular implantable electronic devices (CIEDs) such as pacemakers and implantable cardioverter-defibrillators (ICDs). In a recent case report, a magnetic interaction of the novel iPhone 12 Max with Mag Safe technology, resulting in the therapy inhibition of an ICD *in vivo*, was described for the first time [2]. This magnetic interferaction could be confirmed *in vivo* as well as *ex vivo* in numerous CIEDs [3], [4], [5]. Little is known about the magnetic interaction of smartwatches with CIEDs [6].

This risk of magnetic interaction might become more relevant and challenging in future with the availability of various other small PEDs with integrated magnets, such as electronics cigarettes [7], or the plethora of accessories, such as magnetic wristbands or smartphone covers. Whereas PED market leaders usually indicate on the risk of interactions [8], PED accessories manufacturers and copycat producers of similar products might not even be aware of the inherent risk of their products.

The aim of the current study was to quantify the magnetic fields of latest generation smartwatches using a fast, commercially available accurate field measurement device and to evaluate the relationship between measured field and CIED interaction. Furthermore, a comprehensive summary/overview on other PEDs and their risk of interaction with ICDs is presented.

## 2 Methods

We investigated five smartwatches able to record an intelligent ECG: Apple Watch, Series 6 and Series 7 (Apple Inc, Cupertino, California, USA), Fitbit Sense (Fitbit Inc, San Francisco, CA, USA), Samsung Galaxy Watch 3 (Samsung Inc, Seoul, South Korea), Withings ScanWatch (Withings SA, Issy les Moulineaux, France). All devices are approved by the Food and Drug Administration (FDA) and are CE (Conformité Européene) marked. These devices were selected since they are easily commercially available. Furthermore, a magnetic wristband for the Apple Watch 7 was characterized. Measurements were compared to other PEDs, namely AirPods Pro (opened and closed), the Microsoft Surface Pen, the Apple Pencil 2nd generation, and the iPhone 12 Pro Max.

### 2.1 Magnetic field characterization

**Fig. 1:**
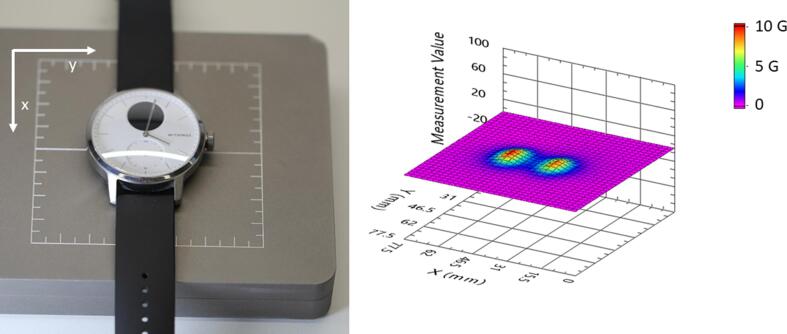
Representative picture of a smartwatch (Withings ScanWatch Model HWA09 42 mm) under test. The watch is placed on the HallinSight® magnetic field camera which displays the field strength in G with a spatial resolution of 2.5 mm along × and y directions. The sensing area of the HallinSight is located 2.5 mm underneath the surface of the back side of the smartwatch.

To characterize the magnetic field strength near the PEDs, measurements with Hall effect sensors were performed. These sensors are available in monolithic three axis versions, allowing to measure the three components of the magnetic field without rotating the probe, which is cumbersome and error-prone. To achieve a field strength mapping of the entire volume near a PED, numerous three axis magnetic field measurements are required. To reach high spatial resolution and avoid the displacement of a single probe at many locations near the PED, a magnetic sensors array called magnetic field camera (MFC) (HallinSight®, Metrolab SA, Switzerland) has been used (Fig. 1). This MFC consists of an array of 32 × 32 = 1024 calibrated three axis Hall sensors with a spatial resolution of 2.5 mm and covering an area of 80 × 80 mm^2^. Measurement points are located with a position error of less than 50 µm and exhibits a magnetic resolution of 4 µT and an amplitude error of 0.1 %. To obtain the magnetic field map, the PEDs have been placed at different heights above the magnetic field camera using simple spacers which were measured with a digital calliper. The measurement data were processed with an in-house algorithm that performs the 3D plot of the 1 mT isogauss lines as well as the calculation of the volume (1 mT volume), which delimits the region surrounding the PED in which the field strength is equal to or greater than 1 mT. The algorithm also calculates the maximal distance at which 1 mT can be observed. We used the 1 mT (=10 G) as cut-off for our analysis based on the ISO standard 14,117 (which is as well recognized by the FDA), requiring a minimal field strength of 1 mT for CIEDs to trigger to magnet mode.

### 2.2 Ex vivo assessment of magnetic interactions

We performed *ex vivo* measurements of the interaction distance between five representative CIEDs with the smartwatches to assess a minimal safety distance (MSD) at which mode switch can be observed. We included three intravenous ICDs from Boston Scientific (Inogen CRT-D, Cognis CRT-D, Teligen ICD) and two from Medtronic (Viva Quad XT CRT-D, Protecta ICD). In addition, a subcutaneous ICD was analysed (Emblem, Boston Scientific). Each smartwatch was approached to the CIEDs at different location of the device three times using a non-magnetic adjustable laboratory lifting platform to identify the distance at which acoustic mode switch was noticeable.This distance was measured using a calliper gauge. Since our aim was to focus on safety based on the minimal safety distance, no drop-out distance at which the magnetic mode is deactivated when removing the device was assessed.

### 2.3 Predictors for magnetic mode switch and interaction distance

To identify predictors for a mode switch of CIEDs due to magnetic interaction, we pooled previously and current measurements of representative and widely available PEDs. In addition to the five smartwatches and one wristband, the wireless charging case of the Apple AirPods Pro (opened and closed), the Microsoft Surface Pen, the Apple Pencil 2nd generation, and the iPhone 12 Pro Max were included, resulting in 55 measurements for the five intravenous CIEDs. Since the MSD between the tested CIEDs was not statistically different, no device selective analysis was performed. In a second analysis, we calculated the predictors for the minimal safety distance based on the assessed field parameters.

### 2.4 Statistical analysis

Continuous variables are presented as mean ± standard deviation (SD) and categorical parameters as frequencies and percentages. Comparisons were made using Student’s *T*-test, or Mann-Whitney *U* test, as appropriate for continuous variables. Normality distribution was assessed using the Kolmogorov-Smirnov test. Discrete variables were compared using Fisher’s exact test. Pearson’s correlation coefficient analysis was performed to assess correlations between the measures (1 mT distance and volume) and the MSD. A linear regression was performed to identify the association between magnetic field measurements and CIED minimal safety distance (MSD). A multivariate linear regression model was developed to assess the risk of magnetic interferaction of the PED with the CIED based on the assumption of a minimal skin thickness of 10 mm. All calculations were performed using SPSS (version 22.0, SPSS Inc., Chicago, ILL) with a p-value < 0.05 considered statistically significant.

## 3 Results

### 3.1 Smartwatch characterization using the magnetic field mapper

**Fig. 2:**
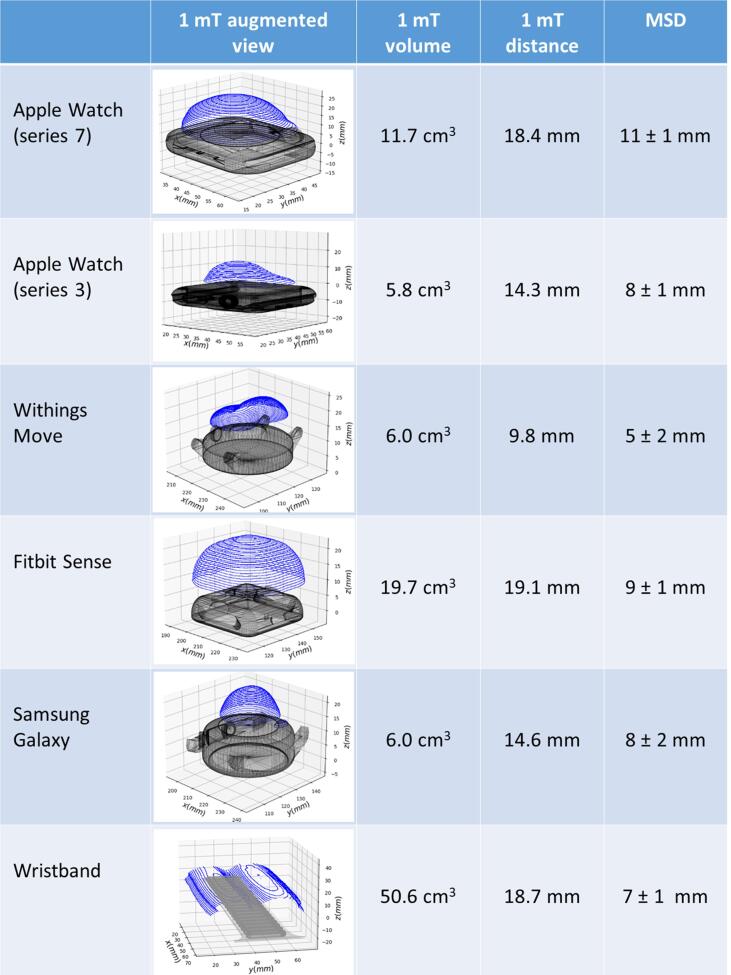
Magnetic field mapping of smartwatches and a wristband using high resolution 3D mapping. The 10 G isogauss lines are displayed in augmented reality together with a 3D view of each device. This highlights a volume where the magnetic field exceeds 1 mT (10 G) and where the CIED shall not penetrate. In the table, the 1 mT volume and and 1 mT distance and the minimal safety distance (MSD) from the ex vivo measurements are listed separately for each smartwatch and a wristband.

All smartwatches could be measured using the device within a few minutes. None of the smartwatches exhibits a maximal field strength > 1 mT while measured from the front-side of the device. On the contrary, all smartwatches exhibit a maximal field strength > 1 mT while measured from the back side. We summarized the magnetic characterization of the five tested popular smartwatches in Fig. 2. In comparison, the 1 mT distance and volume for Apple Watch 7, the iPhone 12 Pro Max and medical ring magnet (St Jude Medical) was 18.4 mm and 11.7 cm^3^, 20.78 mm and 87.4 cm^3^ and 109.7 mm and 3078 cm^3^, respectively.

### 3.2 Ex vivo measurements

The MSD was significantly different between the devices (p < 0.001) ranging from 5 ± 2 mm to 11 ± 1 mm for all CIEDs (Fig. 2). No significant differences in the MSD between the intravenous devices was identified for any of the smartwatches. However, for the subcutaneous ICD (S-ICD), the MSD was lower compared to the intravenous CIEDs (5 ± 3 mm vs 8 ± 2 mm, p = 0.009).

### 3.3 Interaction and predictive modelling

**Fig. 3:**
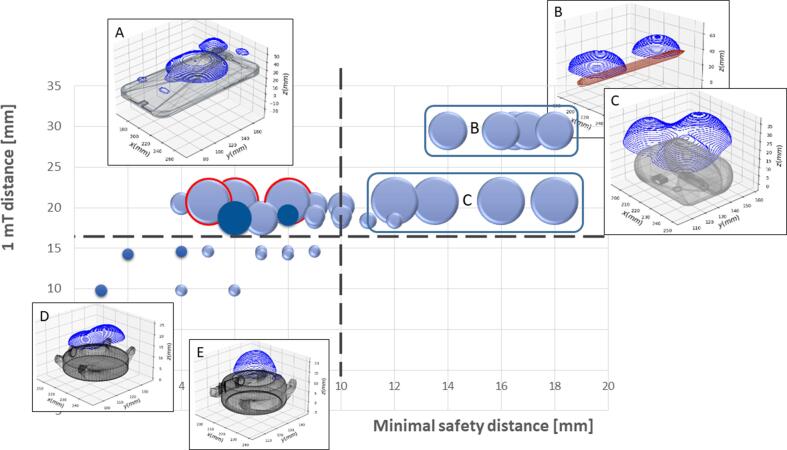
Summary of the maximal 1 mT distance over the minimal safety distance for all CIEDs and PEDs with exemplary augmented 1 mT views of the iPhone (A) (highlighted in red), the Microsoft pen (B), the AirPods Pro charging box (C), the (D) Withings watch, and (E) Samsung watch. The volume of the 1 mT field is represented by the area of the spheres (dark blue: measurements of the 5 smartwatches for the S-ICD; bright blue for the 5 intravenous CIEDs). The augmented reality views of the PEDs with their 1 mT volumes are shown for selective PEDs. *PEDs = Portable electronic devices, CIEDs =* cardiovascular implantable electronic devices, S-ICD = subcutaneous implantable cardioverter defibrillator.

A significant correlation between the MSD as the clinically relevant measures and the 1 mT distance (Pearson rho = 0.632, p < 0.001) as well as the 1 mT volume (Pearson rho = 0.448, p = 0.001) was observed. Furthermore, correlation between the 1 mT distance and volume was identified (Pearson rho = 0.617, p < 0.001). Linear regression analysis showed a linear relationship of the MSD with the 1 mT distance (B coefficient: 0.459; 95 % CI: 0.246–0.672, p < 0.001) but not with the 1 mT volume (B coefficient: 0.005; 95 % CI: −0.050–0.061, p = 0.842). The results for the 1 mT distance (B coefficient: 0.519; 95 % CI: 0.344–0.695, p < 0.001) was confirmed when including all other tested devices (the wireless charging case of the Apple AirPods Pro (open and closed), the Microsoft Surface Pen, the Apple Pencil 2nd generation, and the iPhone 12 Pro Max). This observation is summarized in a comprehensive Fig. 3 for the 11 tested devices with the five intravenous CIEDS. The measurement of the S-ICD is plotted in dark blue and the measures of the iPhone is highlited in red with reported *in vivo* interaction.

Stratification of the MSD based on minimal skin thickness of 10 mm (and consequently a minimal distance of the PED from the CIEDs surface ≥ 10 mm) resulted in a 60 % increase of risk for magnetic mode triggering for every mm increase of the 1 mT distance > 10 mm (OR: 1.603; 95 % CI: 1.110–2.314; p = 0.012).

## 4 Discussion

In this comprehensive study we assessed quantitatively the magnetic field strength and risk of interaction of smartwatches and other PEDs with CIEDs. The main observations are as follows: 1) A great variation in the 1 mT distance and volume, below/above which CIED interferaction is expected to be triggered, was observed for the smartwatches. However, all these measures and interactions were observed only for the back side of the smartwatches. 2) A significant difference was observed for the S-ICD compared to the intranvenuous ICDs, demonstrating a lower sensitivity of the S-ICD on external magnetic fields. 3) With every increase of the 1 mT maximal distance in mm, the minimal safety distance at which a CIED interaction is expected increases by 60 %. With that information, a cut-off of 1 mT at 10 mm might be advisable. 4) For all the tested devices, the relevant distance at which interactions with CIEDs were expected (1 mT maximal distance) and are observed in vitro (MSD) are far below the distance of 6 in. or 18 cm recommended by the FDA [9]. Whether this holds true as well in PED accessories and copycat manufacturers of these and other products [7], however, remains unclear but could benefit from the herein presented measurement technique of the magnetic field. Since not all manufacturers of such products provide appropriate warnings in their user manuals for CIED carriers, heart rhythm specialists should be aware of this possible interaction. Thus, the herein presented results are of clinical relevance and provide guidance for heart rhythm specialists to inform their patients about the estimated risk of interaction of a PED with CIEDs. Permanent magnets are implemented in PED for various reasons. Unlike for some applications as the fixation of the connection with other electronic devices for instance for charging purpose of the phones or smartwatches, the implementation of magnets in other devices such as electronic cigarettes is not obvious. This poses the risk of unawareness of the potential magnetic interactions with CIEDs. In detail, magnetic field strength above 1 mT triggers pacemakers to an asynchronouos pacing mode and results in therapy suspension of ICDs. Whereas the effect of fixed pacing might be sensed by the patients, the deactivation of an ICD might be undetected. This holds true especially in devices without audible warning tone.

The results of our comprehensive comparison of five available smartwatches being capable of acquiring a single-lead ECG are in line with the sparsely available studies on this topic. In a recent study [10], the magnetic field strength was measured to be at 39.2 G, reflecting 3.9 mT, at a distance of 11 mm and 7.8 Gauss at 21 mm from the backside of the Apple watch 7. The field strength of the iPhone 12 Pro Max was measured to be 15.4 G at 11 mm and 7.8 G at 21 mm. Distances enabling a precise location of the potentially relevant 1 mT = 10 G field distance was not reported. The herein observed 1 mT for the Apple watch 6 and 7 of 18.4 mm is well within their reported range. For the iPhone 12 Pro Max, we observed in a previous work [11], a slightly higher 1 mT distance than expected from their study (1 mT at 21 mm compared to their 0.78 mT), which might as well be explained by the more precise 3D measurement obtained with the MFC testing device. Interestingly the iPhone 12 Pro Max, which set the ball rolling for this topic of *in vivo* CIED interaction [2], showed a relatively low in vitro MSD of roughly 8 mm despite the 1 mT distance of 21 mm. This low MSD below 10 mm was confirmed as well by Lacour et al. [4] in numerous other CIEDs, resulting *in vivo* in activation of the magnetic switch in 14 % of the tested CIEDs. Consequently, the cut-off at a 1 mT distance for risk assessment of a device might be appropriate to account for a slim person with low implantation depth of the CIEDs. In practice, with the recommendation of the FDA to keep the device at least 18 cm away from device, the patient is on safe grounds.

### 4.1 Value and implication of the measurement device

In contrast to the previously used single-probe measurement device [12], the herein used device consists of a recently commercially available magnetic field camera intrinsically integrating 1024 three axis sensors. This provides a higher spatial resolution and faster measurements as it does not require to perform the displacement of the sensors along x and y directions. Until now magnetic fields of PEDs were mainly displayed as color maps showing the intensity of the magnetic field at a given distance to the PEDs [11] or even simply as a single point measurement reporting the maximal field strength observed at the surface of the PEDs [10]. To account for the complex geometries of the magnetic fields which is specific to every magnet geometry and magnetization, we propose to display in 3D the 1 mT isogauss lines, resulting in what we call the 1 mT volume. This is the relevant information to estimate the risk of interaction of a PED with CIEDs. The estimation of this volume which is surrounding the PED and where the magnetic field exceeds the 1 mT threshold is to our understanding the best way to assess the probability of interaction of PEDs with CIEDs. In a recent study, we presented an easy-to-use handheld magnetic safety camera that can simplify the magnetic risk assessment of any PED in the clinical setting by physicians [13].

### 4.2 Clinical implication

We determined a relationship between the measured magnetic field parameters and their risk for deactivating ICDs*.* Beside the field strength, the volume of the relevant 1 mT field plays an important role to predict interaction. With a standardized magnetic field camera as the herein presented, the risk assessment is feasible, keeping the “curse” of uncontrollable magnetic fields in PEDs in hand.

This study highlights the importance of public awareness regarding an interaction between smartwatches and CIEDs. Although there seems to be no imminent health risk for CIED patients with using PEDs, certain precautions may be advisable such as avoiding placing the smartwatch directly over the device or in a breast pocket. Recently, work has been published picking up on the possibility of bipolar precordial leads generated through smartwatch recordings [14], [15]. When doing so, clinicians need to be aware of the possible consequences related to placing the watch with its backside on a bare chest.

The built-in magnetic switch sensor in the CIEDs show a hysteresis behaviour, reflected in differences in the distance when the magnetic mode is activated during the approach and deactivated when moving away the device.

### 4.3 Limitation

We investigated only a selection of CIEDs from two companies, focussing on the ones with audible feedback. However, the characteristic of mode switch should not be different between PM and ICD if same sensors are applied and their location within the device is comparable. This was confirmed in the comprehensive study by Lacour et al. [4] showing only negligible difference in the pull-out differences between the devices, concentrating all around a distance of 4 mm. The observed difference might not be clinically relevant in assessing the risk of daily-life magnetic interferaction.

## 5 Conclusion

Whereas the tested smartwatches pose only a negligible risk for magnetic interaction with CIEDs, a standardized magnetic field measurement might help to assess the risk of interaction. The herein used technology has the potential to allow for an individual assessment.

## Funding source

None.

## Declaration of Competing Interest

The authors declare that they have no known competing financial interests or personal relationships that could have appeared to influence the work reported in this paper.

## References

[1] Hindricks G., Potpara T., Dagres N., Arbelo E., Bax J.J., Blomström-Lundqvist C. (2020). 2020 ESC Guidelines for the diagnosis and management of atrial fibrillation developed in collaboration with the European Association of Cardio-Thoracic Surgery (EACTS). Eur. Heart J..

[2] Greenberg J.C., Altawil M.R., Singh G. (2021). Letter to the Editor—Lifesaving therapy inhibition by phones containing magnets. Heart Rhythm..

[3] Nadeem F., Garcia A.N., Tran C.T., Wu M. (2021). Magnetic interference on cardiac implantable electronic devices from apple iphone magsafe technology. J. Am. Heart Assoc..

[4] Lacour P., Dang P.L., Heinzel F.R., Parwani A.S., Bähr F., Kucher A., Hohendanner F., Niendorf T., Rahimi F., Saha N., Han H., Rubarth K., Sherif M., Boldt L.-H., Pieske B., Blaschke F. (2022). Magnetic field–induced interactions between phones containing magnets and cardiovascular implantable electronic devices: flip it to be safe?. Heart Rhythm.

[5] Patterson Z., Straw S., Drozd M., Paton M.F., Cole C., Witte K.K., Gierula J. (2021). To the Editor—New phones, old problem? Interference with cardiovascular implantable electronic devices by phones containing magnets. Heart Rhythm..

[6] Asher E.B., Panda N., Tran C.T., Wu M. (2021). Smart wearable device accessories may interfere with implantable cardiac devices. HeartRhythm Case Rep..

[7] Shea J.B., Aguilar M., Sauer W.H., Tedrow U. (2020). Unintentional magnet reversion of an implanted cardiac defibrillator by an electronic cigarette. HeartRhythm Case Rep..

[8] Apple. About potential magnetic interference with medical devices – Apple Support (UK) [Internet]. [cited 2022 Jan 23];Available from: https://support.apple.com/en-us/HT211900.

[9] FDA U.S. Food and Drug Administration. Magnets in Cell Phones and Smart Watches May Affect Pacemakers and Other Implanted Medical Devices [Internet]. FDA. 2021 [cited 2022 Jan 23]; Available from: https://www.fda.gov/radiation-emitting-products/cell-phones/magnets-cell-phones-and-smart-watches-may-affect-pacemakers-and-other-implanted-medical-devices.

[10] Seidman S.J., Guag J., Beard B., Arp Z. (2021). Static magnetic field measurements of smart phones and watches and applicability to triggering magnet modes in implantable pacemakers and implantable cardioverter-defibrillators. Heart Rhythm.

[11] Quirin T., Féry C., Nicolas H., Madec M., Hébrard L., Knecht S., Pascal J. (2021). Quantification of the safety distance between icds and phones equipped with magnets. JACC: Clin. Electrophysiol..

[12] Han H., Moritz R., Oberacker E., Waiczies H., Niendorf T., Winter L. (2017). Open source 3D multipurpose measurement system with submillimetre fidelity and first application in magnetic resonance. Sci. Rep..

[13] Quirin T., Vergne C., Féry C., Badertscher P., Nicolas H., Mannhard D., Osswald S., Kühne M., Sticherling C., Madec M., Hébrard L., Knecht S., Pascal J. (2022). A magnetic camera to assess the risk of magnetic interaction between portable electronics and cardiac implantable electronic devices. IEEE Int. Symposium Medical Measurements Applications (MeMeA).

[14] Cobos Gil M.Á. (2020). Standard and precordial leads obtained with an apple watch. Ann Intern Med..

[15] Spaccarotella C.A.M., Polimeni A., Migliarino S., Principe E., Curcio A., Mongiardo A., Sorrentino S., De Rosa S., Indolfi C. (2020). Multichannel electrocardiograms obtained by a smartwatch for the diagnosis of ST-segment changes. JAMA Cardiol..

